# Distribution patterns and prediction of lymph, hematopoietic and related tissue cancer incidence in Chinese cancer registration areas from 2005 to 2050

**DOI:** 10.3389/fonc.2026.1919295

**Published:** 2026-08-10

**Authors:** Lijun Lu, Xin Pan, Xiaohong Chen, Weixia Nong

**Affiliations:** 1 Department of Hematology, The First Affiliated Hospital of Shihezi University, Shihezi, Xinjiang, China; 2 National Hematology Clinical Research Center Xinjiang Production and Construction Corps Branch Center, Shihezi, Xinjiang, China

**Keywords:** cancer registration, forecasting research, incidence, lymph, hematopoietic and related tissue cancer, regression analysis, spatial distribution

## Abstract

**Objective:**

By analyzing the spatiotemporal distribution of lymph, hematopoietic and related tissue cancer incidence in cancer registration areas of China from 2005 to 2019 and predicting future trends, this study provides references for the prevention and control of related cancers in China.

**Methods:**

The data on lymph, hematopoietic and related tissue cancer (ICD-10, C81-C85, C88, C90, and C96) incidence were from the China Cancer Registry Annual Report. The Joinpoint regression model was employed to calculate the average annual percentage change (AAPC) for describing time trends, the age-period-cohort model was used to analyze age, period, and cohort effects on incidence, the Bayesian age-period-cohort (BAPC) model was used to predict the incidence from 2020 to 2050, and the spatial autocorrelation methods was used to characterize spatial distribution patterns.

**Results:**

From 2005 to 2019, the lymph, hematopoietic and related tissue cancer incidence in Chinese cancer registry regions was 6.39/100,000, with an age-standardized incidence rate (ASIR) of 4.17/100,000. During this period, ASIR showed an upward trend, with an AAPC of 1.2% (95% CI: 0.6%, 1.8%). The incidence and ASIR were higher in males than in females, and higher in urban than in rural areas. However, the upward trend was more pronounced among females and in rural areas. From 2020 to 2050, the ASIR was predicted to continue its upward trend. The incidence risk of lymph, hematopoietic and related tissue cancer increased with age, particularly in the 45–75 age group. Eastern coastal developed regions were high-incidence areas.

**Conclusions:**

From 2005 to 2019, the ASIR of lymph, hematopoietic and related tissue cancer in Chinese cancer registry regions showed an upward trend and continued to increase by 2050. Significant disparities existed across age, gender, and regions. Relevant authorities should maintain close monitoring of lymph, hematopoietic and related tissue cancer distribution patterns, actively identify high-risk populations and areas, and implement targeted measures to reduce the incidence risk of related cancer.

## 1 Introduction

Cancer, as a major chronic disease, poses a substantial threat to human health and survival worldwide. In recent years, its incidence and mortality have continued to increase, not only creating serious public health challenges but also imposing a considerable disease burden and economic pressure on societies and families. According to GLOBOCAN 2022 estimates, there were 19.97 million new cancer cases and 9.74 million deaths worldwide. China accounted for 4.82 million new cancer cases, accounting for 24.2% of the global total and 26.4% of global cancer deaths. Both the incidence and mortality of cancer in China surpassed the global average (1).

Lymphoma ranks among the relatively common cancer. It is essentially a cancer of the immune system originating in lymph nodes and lymphoid tissue, encompassing Hodgkin lymphoma, non-Hodgkin lymphoma, and their subtypes (2). According to the latest estimates (1), there were 635,000 new lymphoma cases worldwide in 2022, with 273,000 deaths due to the disease. In China, there were 85,200 new cases, ranking 15th among all types of cancer, and 41,600 deaths, ranking 13th on the cancer mortality list (3). With the burden of lymphoma increasing annually (4), the prevention and control of lymphoma has become a pressing public health challenge for China.

By analyzing the epidemiological characteristics and temporal trends of the disease, important evidence can be provided for prevention and control efforts. However, previous studies on lymphoma have predominantly focused on the global level, developed nations, or localized regions within China (5–7). Studies offering a multidimensional “person-time-space” description from a national perspective in China remain insufficient. This study comprehensively integrated data released by the China Cancer Registry Annual Report from 2008 to 2022, covering nationwide incidence information on lymph, hematopoietic and related tissue cancer stratified by age, sex, and region. To accurately describe incidence trends, the Joinpoint regression model was applied to quantify temporal variations in lymph, hematopoietic and related tissue cancer incidence. Subsequently, the age-period-cohort model was used to decompose the independent contributions of age, period, and cohort effects to incidence risk. To predict future incidence trends, the Bayesian age-period-cohort (BAPC) model was applied. Spatial autocorrelation analysis was also applied to explore geographical distribution patterns of cases. This study provided empirical evidence for formulating lymph, hematopoietic and related tissue cancer prevention and control policies in China, laying a robust data foundation for precision prevention and treatment of this disease.

## 2 Methods

### 2.1 Data sources

Data on the incidence and age-standardized incidence rate (ASIR) of lymph, hematopoietic and related tissue cancer in China from 2005 to 2019 were derived from the China Cancer Registry Annual Report, 2008-2022. Lymph, hematopoietic and related tissue cancer cases in this study were selected based on the International Classification of Diseases, Tenth Revision (ICD-10) (8), with inclusion codes of C81–C85, C88, C90, and C96; specifically, C81 represents Hodgkin lymphoma, C82–C85 and C96 represent Non-Hodgkin lymphoma, C88 represents Immunoproliferative diseases, and C90 represents multiple myeloma. According to the China Cancer Registry Annual Report, prefecture-level and higher administrative divisions were classified as urban areas, while counties and county-level cities were classified as rural areas.

### 2.2 Joinpoint regression model

The Joinpoint regression model is a statistical tool used to describe the continuous trend of disease incidence over time. It is capable of evaluating trend changes across different periods throughout the entire time span. This model creates segmented regressions based on the time characteristics of disease distribution. It divides the study period into distinct intervals using multiple joinpoints, then fits and optimizes the trend within each interval to assess the characteristics of specific disease changes across different periods. The model determines the number of breakpoints, their positions, and corresponding *P*-values (two-tailed test, significance level α=0.05) using Monte Carlo permutation testing. When analyzing the continuous trend of cancer incidence in a population, the log-linear model is typically employed, expressed as (9):


$$\text{E}\left[\text{y}/\text{x}\right]={\text{e}}^{\beta_{0}+\beta_{1}\text{x}+\delta_{1}{\left(\text{x}-\tau_{1}\right)}^{+}+\cdots +\delta_{\text{k}}\left(\text{x}-\tau_{\text{k}}\right)}$$

In the formula, e is the base of the natural logarithm, k is the number of inflection points, τ_k_ is the number of unknown inflection points, β_0_ is the constant parameter, β_1_ is the regression coefficient, δ_k_ is the slope of the kth segmental function.

Further, the annual percentage change (APC) was used to assess the trends within each subinterval, the average annual percentage change (AAPC) was used to evaluate the overall trend, and the statistical significance was determined based on the 95% confidence interval (95% CI). When the number of turning points is zero, APC=AAPC. When APC>0 or AAPC>0 and its 95% CI>0, it indicates a significant upward trend during that period; conversely, a significant downward trend is indicated.

### 2.3 Age-period-cohort model

This study applied the age-period-cohort model to quantify the independent effects of age, period, and cohort on incidence, revealing the role of demographic shifts in driving disease trends and estimating the magnitude of each factor’s effect, as expressed by the formula (10, 11):


$$\log\left(\lambda_{\text{apc}}\right)=\alpha_{\text{a}}+\beta_{\text{p}}+\gamma_{\text{c}}+\epsilon$$

In the formula, α, β and γ are the effects of age, period, and cohort, respectively, while ϵ is the residual. The relative risk (RR) of incidence was used to describe trend changes, where RR<0 indicates a reduced risk of incidence, and RR>0 indicates an increased risk.

### 2.4 Bayesian age-period-cohort model

Using the BAPC model to predict the incidence of lymph, hematopoietic and related tissue cancer in China over the next 30 years. In this model, a second-order stochastic walk model is applied to smooth prior age, period, and cohort effects for predicting posterior incidence. Our method combines ensemble nested Laplace approximation to prevent mixing and convergence issues caused by Markov chain Monte Carlo sampling, thereby yielding more stable and reliable data. The formula is (12):


$$f(\alpha |k_{\alpha })\propto k_{\alpha }^{\frac{I-2}{2}}\exp(-\frac{k_{\alpha }}{2}\sum_{i=3}^{I}{\left(\alpha_{i}-2\alpha_{i-1}+\alpha_{i-2}\right)}^{2})=k_{\alpha }^{\frac{I-2}{2}}\exp(-\frac{1}{2}\alpha^{T}Q\alpha )$$

Here, *k* is the precision parameter, Q is the rank, and *α_i_* (*i* = 1, …, *I*) is the age effect.

### 2.5 Spatial autocorrelation analysis

This study employed ArcGIS software to analyze lymph, hematopoietic and related tissue cancer case data at the provincial level using spatial autocorrelation analysis. The analysis encompasses two methodologies: global spatial autocorrelation and local spatial autocorrelation (13).

Global spatial autocorrelation analysis was adopted to assess the spatial clustering characteristics of lymph, hematopoietic and related tissue cancer incidence overall. Moran’s *I* index (range: -1 to 1) served as the primary metric to reflect the spatial association degree of these cancer cases.

Local spatial autocorrelation analysis focused on finer spatial scales to precisely identify hotspots of lymph, hematopoietic and related tissue cancer incidence. Similarly conducted at the provincial level, it calculates the Local Indicator of Spatial Association to classify spatial clustering patterns into four types: high-high, low-low, high-low, and low-high. Among these, high-high and low-low clustering represent homogeneous clustering, indicating that the observed values in a province are similar to those in surrounding provinces—i.e., the region and its neighbors share either high or low incidence levels. High-low and low-high clustering represent heterogeneous clustering, indicating that the observed values in a province are higher or lower than those in surrounding provinces.

### 2.6 Statistical methods

Joinpoint regression analysis was performed using the Joinpoint Regression Program 4.9.1.0 software developed by the National Cancer Institute. The age-period-cohort model was constructed using the online web-based analysis tool developed by Rosenberg and Check et al (14). The BAPC model was created using the BAPC and INLA packages in R 4.3.1 software to predict the incidence trends of lymph, hematopoietic and related tissue cancer in China from 2020 to 2050 based on relevant data. ArcGIS 10.8 software was used to analyze the spatial distribution characteristics of lymph, hematopoietic and related tissue cancer incidence in China from 2010 to 2016. The significance level was set at *α* = 0.05.

## 3 Results

### 3.1 Incidence and its trends of lymph, hematopoietic and related tissue cancer in Chinese cancer registry regions, 2005-2019

From 2005 to 2019, a total of 230,114 new lymph, hematopoietic and related tissue cancer cases were registered in Chinese cancer registry regions. Among these, 132,990 cases (57.8%) occurred in males and 97,124 cases (42.2%) in females. Urban areas accounted for 138,581 cases (60.2%), while rural areas had 91,533 cases (39.8%). The overall incidence was 6.39/100,000 (7.29/100,000 for males, 5.47/100,000 for females; 7.57/100,000 in urban areas, 5.17/100,000 in rural areas), with an ASIR of 4.14/100,000 (4.88/100,000 for males, 3.44/100,000 for females; 4.73/100,000 in urban areas; 3.25/100,000 in rural areas). Results revealed that male’s incidence and ASIR were higher than females; urban areas incidence and ASIR were higher than rural areas (**Table 1**).

**Table 1:** Incidence of lymph, hematopoietic and related tissue cancer in Chinese cancer registry regions, 2005-2019 (1/100,000).

Year	National			Male			Female			Urban			Rural		
	Case	Rate	ASIR	Case	Rate	ASIR	Case	Rate	ASIR	Case	Rate	ASIR	Case	Rate	ASIR
2005	3381	6.16	3.71	1990	7.15	4.47	1391	5.13	2.98	2880	7.08	4.16	501	3.52	2.39
2006	3829	6.43	3.93	2262	7.54	4.71	1567	5.30	3.21	3328	7.15	4.29	501	3.85	2.68
2007	3625	6.06	3.57	2102	6.95	4.17	1523	5.15	3.01	3026	6.78	3.91	599	3.94	2.64
2008	4771	7.12	4.04	2759	8.28	4.74	2012	6.13	3.37	4188	8.03	4.45	583	4.17	2.52
2009	5713	6.68	3.75	3332	7.71	4.46	2381	5.64	3.05	4718	8.21	4.47	995	3.56	2.18
2010	7427	5.96	4.31	4387	6.96	5.18	3040	4.94	3.48	5833	7.29	5.11	1594	3.57	2.76
2011	8888	6.1	4.39	5187	7.04	5.21	3701	5.13	3.60	6389	7.3	5.01	2499	4.29	3.35
2012	11351	5.73	4.12	6510	6.49	4.80	4841	4.96	3.47	7222	7.19	4.89	4129	4.23	3.25
2013	13984	6.17	4.32	8133	7.08	5.11	5851	5.24	3.55	8559	7.67	5.05	5425	4.72	3.53
2014	17331	6.01	4.19	10043	6.87	4.89	7288	5.13	3.51	10532	7.31	4.85	6799	4.72	3.47
2015	20655	6.44	4.41	12042	7.4	5.18	8613	5.45	3.66	11719	7.6	4.98	8936	5.36	3.84
2016	24145	6.33	4.30	13887	7.17	4.98	10258	5.46	3.62	14314	7.43	4.87	9831	5.2	4.67
2017	28180	6.46	4.34	16268	7.36	5.07	11912	5.54	3.63	16099	7.55	4.91	12081	5.43	3.78
2018	34440	6.58	4.38	19748	7.44	5.07	14692	5.7	3.70	18107	7.67	4.91	16333	5.69	3.91
2019	42394	6.75	4.38	24340	7.63	5.09	18054	5.83	3.69	21667	8.07	5.11	20727	5.76	3.82
Total	230114	6.39	4.14	132990	7.29	4.88	97124	5.47	3.44	138581	7.57	4.73	91533	5.17	3.25

ASIR, age-standardized incidence rate.

**Figure 1:**
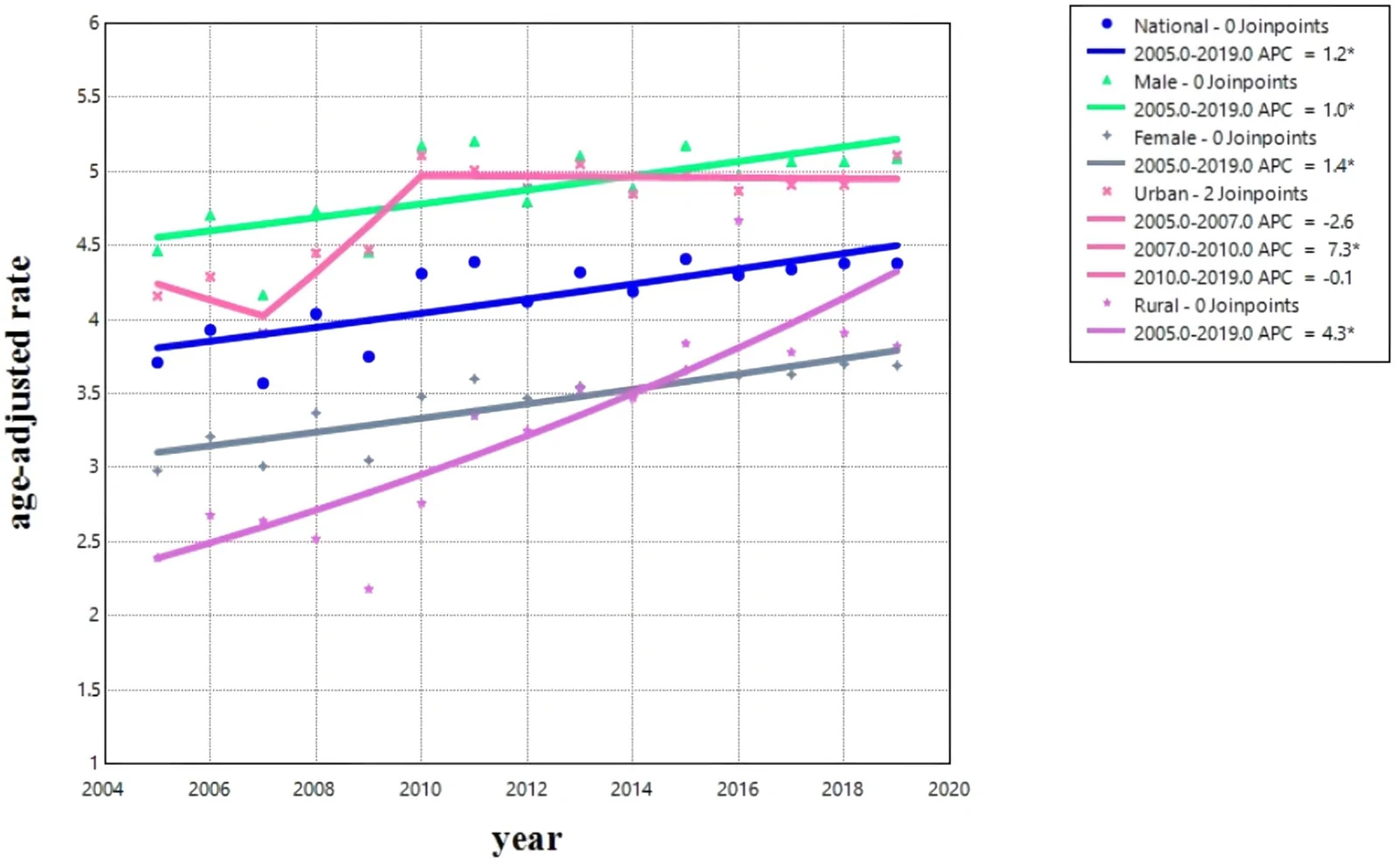
Incidence trends of lymph, hematopoietic and related tissue cancer in Chinese cancer registry regions, 2005-2019. APC, annual percent change. *Indicates statistically significant difference, P<0.05.

From 2005 to 2019, the ASIR increased from 3.71/100,000 to 4.14/100,000, with an AAPC of 1.2% (95% CI: 0.6%, 1.8%). For males, the ASIR increased from 4.47/100,000 to 4.88/100,000, with an AAPC of 1.0% (95% CI: 0.3%, 1.6%). For females, it increased from 2.98/100,000 to 3.44/100,000, with an AAPC of 1.4% (95% CI: 0.9%, 2.0%). By region, the ASIR in urban areas rose from 4.16/100,000 to 4.73/100,000, with an AAPC of 1.1% (95% CI: -0.4%, 2.6%), but this increase was not statistically significant. This growth included three time periods: from 2005 to 2007, there was a downward trend but it was not statistically significant, with an APC of -2.6% (95% CI: -9.1%, 4.4%); from 2007 to 2010, there was an upward trend, with an APC of 7.3% (95% CI: 0.3%, 14.8%); and from 2010 to 2019, there was a downward trend but it was not statistically significant, with an APC of -0.1% (95% CI: -0.6%, 0.5%). In rural areas, it rose from 2.39/100,000 in 2005 to 3.25/100,000 in 2019, with an AAPC of 4.3% (95% CI: 2.8%, 5.9%) (**Figure 1**).

### 3.2 Age-period-cohort analysis

Nationwide analyses across genders and urban-rural settings revealed a highly consistent trend in the age-related incidence of lymph, hematopoietic and related tissue cancer. To be specific, among individuals aged 0-45, the incidence risk increased gradually with age. Between the ages of 45-75, the risk grew rapidly with age. After age 75, the risk began to decline with age.

Period effect analysis revealed stable trends in lymph, hematopoietic and related tissue cancer incidence nationwide, among males, and in urban areas. Conversely, incidence among females and rural areas showed an upward trend after 2010, particularly in rural areas.

**Figure 2:**
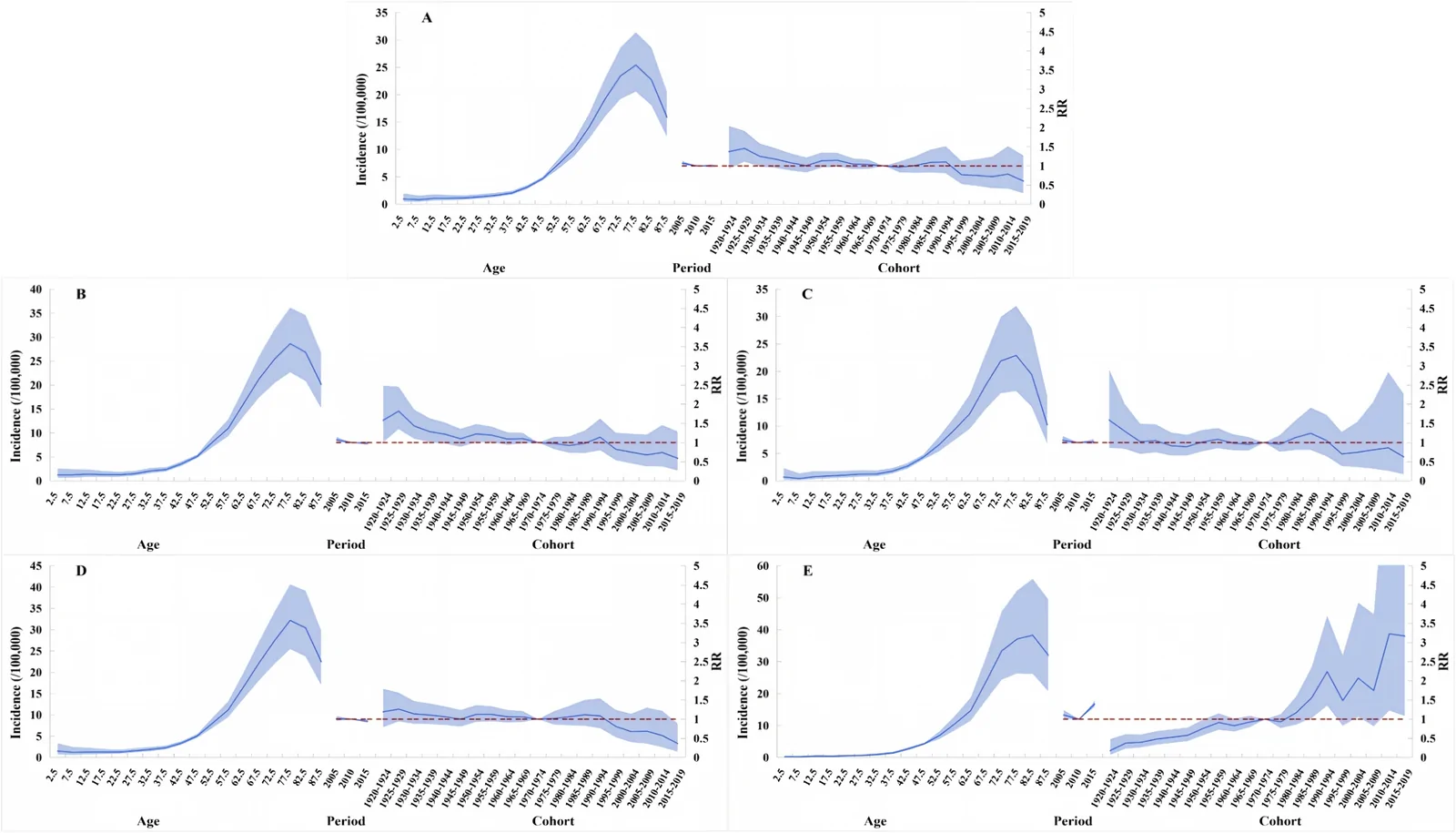
Age-period-cohort analysis of lymph, hematopoietic and related tissue cancer incidence in Chinese cancer registry regions, 2005-2019. National **(A)**, males **(B)**, females **(C)**, urban areas **(D)**, and rural areas **(E)**.

From the cohort perspective, birth cohorts prior to 1970–1974 exhibited higher nationwide incidence risk than subsequent cohorts. Further analysis revealed that the cohort effect trends for lymph, hematopoietic and related tissue cancer incidence risk among males, females, and urban areas were consistent with the national patterns. However, rural areas exhibited a distinct pattern: cohort groups born after 1970–1974 not only showed a higher cohort effect for incidence risk compared to earlier cohorts but also demonstrated a sustained upward progression overall (**Figure 2**).

### 3.3 Prediction of lymph, hematopoietic and related tissue cancer incidence in Chinese cancer registry regions

**Figure 3:**
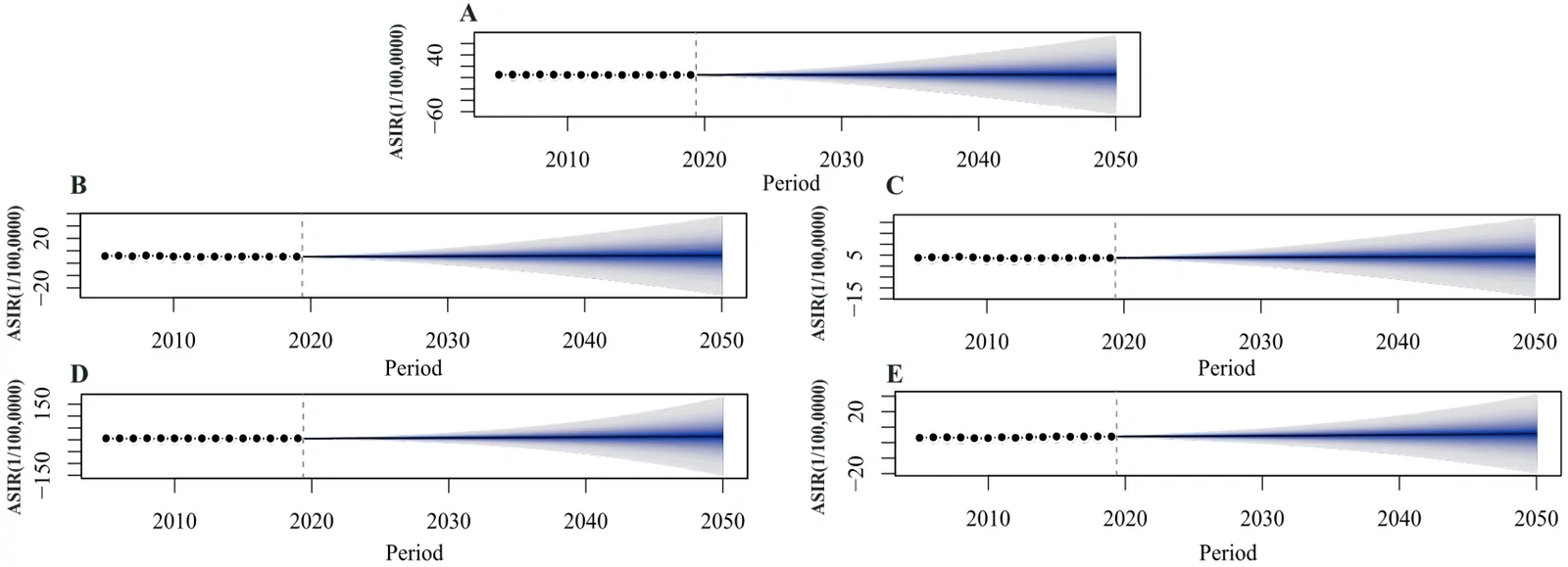
Prediction incidence of lymph, hematopoietic and related tissue cancer in Chinese cancer registry regions from 2020 to 2050. National **(A)**, urban areas **(B)**, rural areas **(C)**, males **(D)**, and females **(E)**.

According to the BAPC model predictions, the lymph, hematopoietic and related tissue cancer ASIR will show a sustained upward trend between 2020 and 2050. Predictions indicated that male incidence will remain higher than female incidence, rising from 5.13/100,000 and 3.74/100,000 in 2020 to 6.10/100,000 and 4.21/100,000, respectively. Furthermore, predictions indicated that urban incidence would remain higher than rural incidence in the future, rising from 5.31/100,000 and 3.85/100,000 in 2020 to 14.33/100,000 and 5.73/100,000 in 2050, respectively (**Figure 3**).

### 3.4 Spatial autocorrelation analysis of lymph, hematopoietic and related tissue cancer incidence in Chinese cancer registry regions, 2010-2016

**Figure 4:**
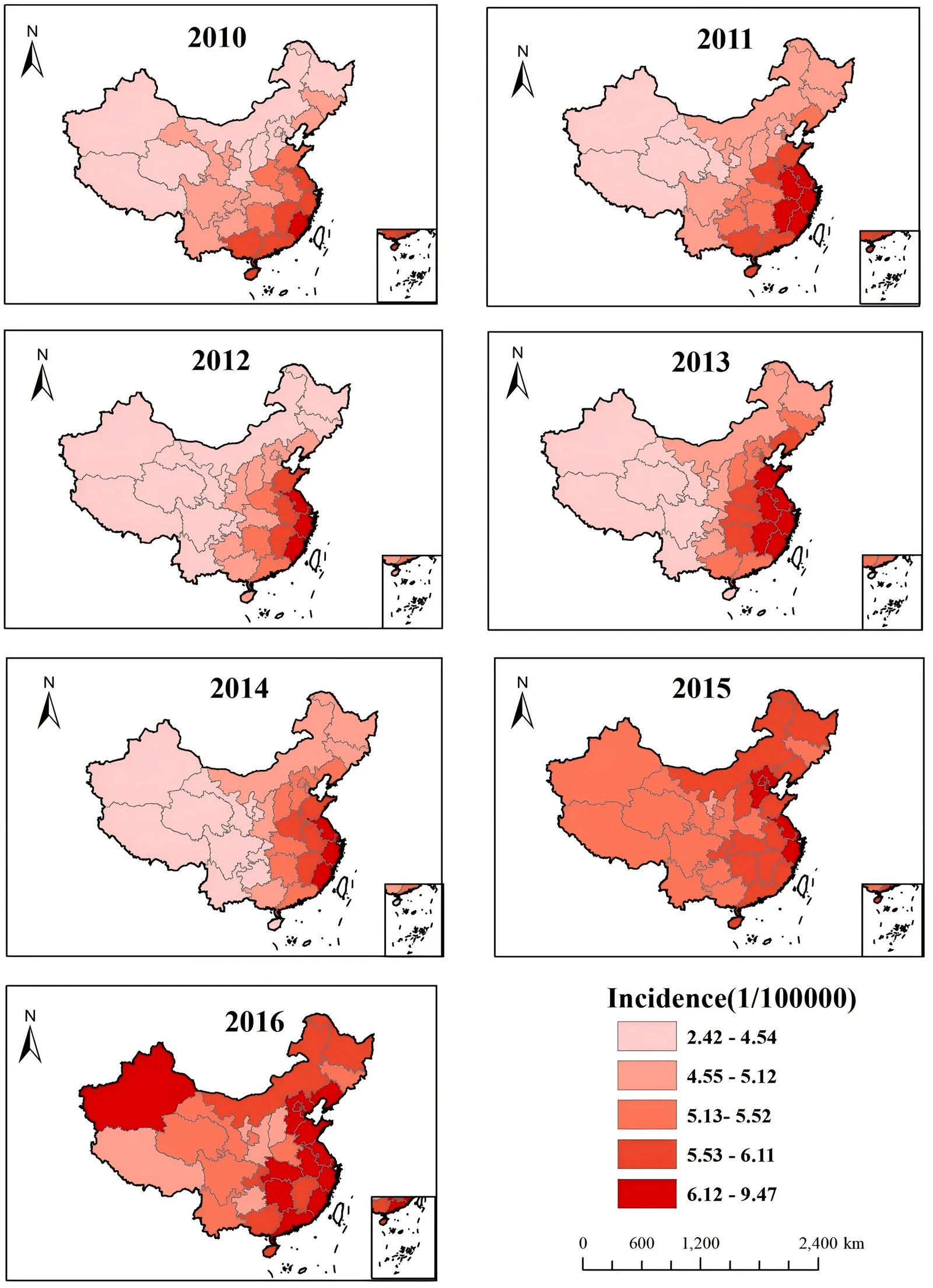
Lymph, hematopoietic and related tissue cancer incidence in different regions in Chinese cancer registry regions, 2010-2016. Map from the Ministry of Natural Resources of China, cartographic license: GS (2019) 182.

During the period from 2010 to 2016, lymph, hematopoietic and related tissue cancer incidence increased across all provinces in China, with the highest concentrations observed in the eastern coastal areas, including Zhejiang, Fujian, Jiangsu, and Shanghai (**Figure 4**).

The global spatial autocorrelation analysis of lymph, hematopoietic and related tissue cancer incidence nationwide from 2010 to 2016 revealed that during 2010-2013, Moran’s *I* index fluctuated between -0.115 and 0.088, with corresponding *P*-values consistently exceeding 0.05. This indicated that spatial autocorrelation in lymph, hematopoietic and related tissue cancer incidence was insignificant during this phase, with overall distribution appearing random. From 2014 to 2015, Moran’s *I* rose to 0.117 and 0.130, with Z-values of 2.032 and 2.192 (*P* < 0.05), respectively. This indicated a positive spatial correlation in lymph, hematopoietic and related tissue cancer incidence during these two years, characterized by distinct clustering of high-incidence provinces with other high-incidence provinces and low-incidence provinces with other low-incidence provinces. In 2016, Moran’s *I* index declined to 0.090, with the *P*-value rising to 0.097. The spatial positive correlation lost statistical significance, indicating weakened clustering. The 2015 Moran’s coefficient reached its maximum value, reflecting the highest spatial positive correlation and clustering intensity in lymph, hematopoietic and related tissue cancer incidence nationwide (**Table 2**).

**Table 2:** Global Moran’s *I* index for lymph, hematopoietic and related tissue cancer incidence in Chinese cancer registry regions, 2010-2016.

Year	Moran’s, *I* index	Expectation index	Variance	*Z*	*P*
2010	0.568	-0.033	0.006	7.861	0.000
2011	0.444	-0.033	0.006	6.247	0.000
2012	0.417	-0.033	0.006	5.965	0.000
2013	0.466	-0.033	0.006	6.533	0.000
2014	0.456	-0.033	0.006	6.441	0.000
2015	0.467	-0.033	0.006	6.684	0.000
2016	0.364	-0.033	0.005	5.406	0.000

**Figure 5:**
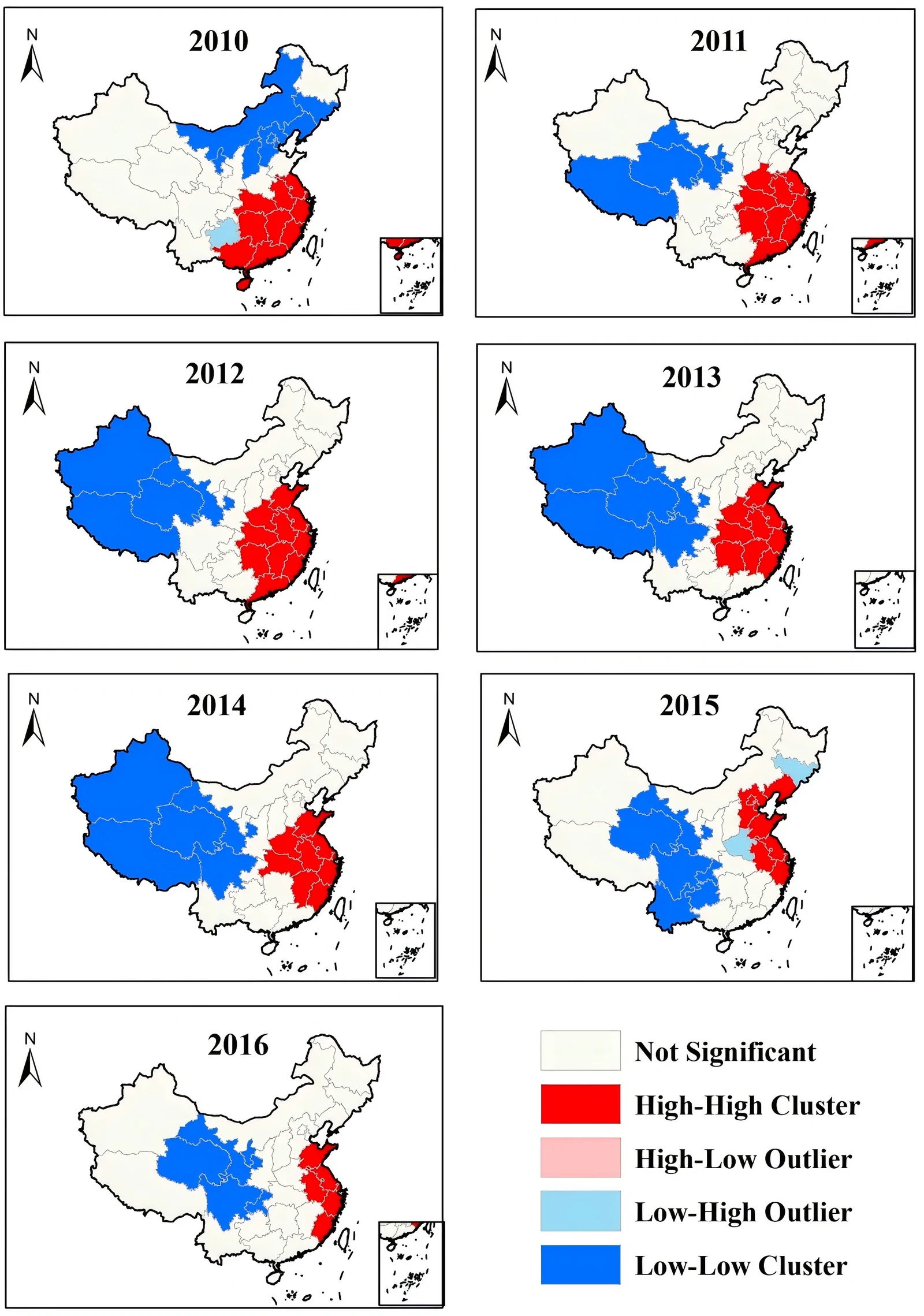
Local spatial autocorrelation of lymph, hematopoietic and related tissue cancer incidence in Chinese cancer registry regions, 2010-2016. Map from the Ministry of Natural Resources of China, cartographic license: GS (2019) 182.

Using ArcGIS software, the local spatial autocorrelation analysis was conducted on the incidence of lymph, hematopoietic and related tissue cancer nationwide from 2010 to 2016. From 2010 to 2016, partial areas exhibited three spatial clustering patterns, including high-high clusters, low-low clusters, and low-high clusters. In 2010, high-high clusters were distributed in the eastern and southern regions. These high-high clusters shifted eastward from 2011 to 2016 and were solely distributed in the eastern regions in 2016. Low-low clusters were located in the northern regions in 2010 and gradually transferred from the northern regions to the western regions during 2010–2016, with low-low clusters concentrated in the western regions in 2016 (**Figure 5**). Overall, the high-high clusters were mainly distributed in the eastern regions. The low-low clusters showed a shifting trend from the northern regions toward the western regions. The low-high cluster was less abnormal.

## 4 Discussion

From 2005 to 2009, the ASIR of lymph, hematopoietic and related tissue cancer in Chinese cancer registry regions showed an upward trend, with noticeable variations across gender, age, and region. This study further revealed that while lymph, hematopoietic and related tissue cancer incidence was higher among males in China, the rate of increase was faster among females. GLOBOCAN data also indicated a global upward trend in lymphoma incidence, particularly among women, younger individuals, and populations from Asian countries (1). Additional studies have similarly demonstrated that the burden of lymphoma was typically higher in males (5, 15). One possible explanation for this gender difference is that overweight and obesity are more prevalent among males (16). Apart from genetic factors and viral infections, the development of lymphoma is closely associated with metabolic risks such as high body mass index (BMI) (17). BMI is also associated with clinical efficacy and survival outcomes in patients with hematologic cancer (18, 19). A cohort study of 5.8 million individuals found a 10% increased risk of Hodgkin lymphoma for every 5kg/m² increase in BMI (20). Obesity represents a state of chronic inflammation, leading to alterations in plasma adipokines including growth factors and pro-inflammatory cytokines, all of which participate in biological processes such as immune responses. Furthermore, elevated BMI correlates with reduced secretion of leptin and adiponectin, potentially contributing to insulin resistance and downstream pathway activation, thereby promoting cancer development. Further studies have indicated that obesity can alter the cancer microenvironment to promote cancer progression, affecting immune cell composition and PD-1 expression (21, 22).

This association may also relate to physiological and hormonal differences between males and females (23). Higher androgen levels in males may exert an immunosuppressive effect, reducing surveillance of abnormal cells and increasing the risk of cancer transformation. Furthermore, androgens may promote abnormal proliferation of certain lymphocyte subsets, creating conditions conducive to cancer development. In contrast, estrogen in females exhibits immune-enhancing effects, potentially improving the body’s ability to clear viral infections and cancer cells. Moreover, numerous other risk factors for lymphoma, such as genetic predisposition (24), physical and chemical agents (25) (e.g., radiation, air pollution), and unhealthy lifestyles (26–28) (e.g., smoking, alcohol consumption, sedentary habits), may be more prevalent among men. For instance, as China’s economy continues to develop and industrialization advances, residents face increasing exposure to lymphoma risk factors. Men, due to occupational factors, may be more likely to be exposed to these hazards (29). Therefore, lymphoma prevention and control strategies should persistently incorporate gender-specific approaches.

The age effect observed in this study indicated that the risk of developing lymph, hematopoietic and related tissue cancer increased rapidly with age among individuals aged 45-75. Another study has shown that lymphoma incidence was significantly higher in those over 65, peaking in the 65–84 age group (15). It is well known that the elderly represent one of the most vulnerable groups for most cancers, facing heightened risks across multiple cancers due to compromised immune function, poor health status, and the cumulative effects of long-term risk factors. This significantly amplifies the challenges posed by the global demographic shift toward aging populations. China has already entered an aging society, with individuals aged 65 and older constituting 13.53% of the population in 2020, and this proportion is projected to exceed 28% by 2040 (30). Therefore, given the trend of population aging in China and globally, it is crucial to incorporate healthy aging strategies into long-term planning. This includes continuously strengthening health promotion programs and community care services to enhance the quality of life for elderly patients.

Numerous studies have indicated that cancer incidence was more severe in economically developed regions (9, 31). Our study found that lymph, hematopoietic and related tissue cancer incidence in China’s urban areas exceeded that in rural areas, although the upward trend was more pronounced in rural areas. A study categorizing global regions based on social development index (SDI, a composite indicator measuring a country’s development level, encompassing per capita income, education levels, and fertility rates to effectively capture socioeconomic factors influencing health outcomes) revealed that lymphoma incidence continued to rise in high-SDI countries and was demonstrably higher than in low-SDI regions. Analysis attributed this to high-SDI countries’ significant advantages in cancer screening, treatment technologies, and drug accessibility, while low-SDI countries face multiple challenges including delayed diagnosis, inadequate treatment, and low survival rates (32). Concurrently, lifestyle changes in developed regions, such as chronic stress and irregular schedules, may compromise immune function and elevate disease risk (33). An additional study has indicated that the 5-year survival rate for Hodgkin lymphoma exceeded 90% in regions with high SDI, whereas it fell below 50% in areas with low SDI (34). Studies have also suggested that urban populations face greater exposure to multiple lymphoma risk factors compared to rural residents. These included pollution from home renovations, psychological stress stemming from fast-paced lifestyles, prolonged exposure to ionizing radiation environments, and air quality issues (35, 36). These factors may collectively contribute to the urban–rural disparities in lymphoma incidence. Spatial autocorrelation analysis in this study also identified Zhejiang, Jiangsu, and Shanghai as high-incidence areas for lymph, hematopoietic and related tissue cancer in China, and these areas are also regions with developed economies in China. The rapid rise in lymphoma incidence in China’s rural areas was consistent with previous findings, such as the lower but gradually increasing disease burden in low- and middle- SDI regions. This trend may be linked to economic development, lifestyle changes, and improvements in healthcare standards (37). In essence, health inequalities observed from this perspective manifest not only in survival rates but also in the geographic distribution and long-term trends of disease burden.

This study has limitations. The data used in this study were obtained from cancer registration programs across various regions of China. Due to their limited coverage, the representativeness of the study population and the generalizability of the findings may be insufficient. The latest cancer statistics reported in the China Cancer Registry Annual Report published by the National Cancer Center have a three-year delay, and the current report is updated to 2019. The study scope encompassed 31 provinces (autonomous regions and municipalities) and the Xinjiang Production and Construction Corps, excluding Hong Kong, Macau, and Taiwan. Future study should integrate data from additional regions to better reflect the national picture. Due to data limitations, this study did not investigate additional risk factors, which limited the comprehensiveness of the findings.

## 5 Conclusion

In summary, based on China Cancer Registry Annual Reports, the ASIR of lymph, hematopoietic and related tissue cancer in Chinese cancer registry regions showed an upward trend from 2005 to 2019, and is predicted to continue rising through 2050. Moreover, differences existed in incidence and their trends between males and females, as well as between urban and rural areas. The eastern region may be a hotspot for lymph, hematopoietic and related tissue cancer incidence. These findings suggested that we must continue to focus on and implement differentiated prevention and treatment strategies for lymph, hematopoietic and related tissue cancer, with particular emphasis on high-incidence areas and high-risk populations.

## Data Availability

The original contributions presented in the study are included in the article/supplementary material, further inquiries can be directed to the corresponding author/s.
